# The effects of if-then plans on weight loss: results of the 24-month follow-up of the McGill CHIP Healthy Weight Program randomized controlled trial

**DOI:** 10.1186/s13063-019-4014-z

**Published:** 2020-01-07

**Authors:** Bärbel Knäuper, Huma Shireen, Kimberly Carrière, Mallory Frayn, Elena Ivanova, Zhen Xu, Ilka Lowensteyn, Gentiana Sadikaj, Aleksandra Luszczynska, Steven Grover, McGill CHIP Healthy Weight Program Investigators, Anaïs Ames-Bull, Shannon Caron, Melodie Chamandy, Farah Islam, Jenna Morris, Constanza Rosemary Lempereur de Saint Pierre, Julia Lévy-Ndejuru, Virginia Rogers, Anna Saint-Martin, Michelle Sasson, Anastasiya Voloshyn

**Affiliations:** 1 Department of Psychology, McGill University, Montreal, QC Canada; 2 University of Toronto, Toronto, Ontario Canada; 3 University of British Columbia, Okanagan Campus, Kelowna, BC Canada; 4 Department of Medicine and Epidemiology and Biostatistics, McGill University, Montreal, QC Canada; 5 SWPS University of Social Sciences and Humanities, Wroclaw Faculty of Psychology, Wroclaw, Poland

**Keywords:** Lifestyle modifications, Weight loss, Behavior modifications, Clinical trials, Diabetes

## Abstract

**Background:**

Current evidence suggests that some of the most effective weight loss approaches are changes in dietary and physical activity behaviors through lifestyle modification programs. The Group Lifestyle Balance (GLB) program is a group-based behavior modification program aimed at changing diet and physical activity for weight loss. It was developed to be more cost-effective and easier to disseminate than its individually administered parent program, the Diabetes Prevention Program (DPP). However, the average weight loss following participation in the GLB is only approximately 3.5%, with low long-term weight loss maintenance.

**Purpose:**

We aimed to optimize the weight loss outcomes of the GLB to increase the efficacy already afforded by its cost-effectiveness and ease of dissemination. We did this by integrating the habit formation tool of if-then plans into the program. This program is called the enriched GLB or the McGill Comprehensive Health Improvement (CHIP) Healthy Weight Program. Results at 3 and 12 months of participation have already been published elsewhere. They showed no between-group differences between the standard and enriched GLB but higher weight loss in both groups compared to the DPP. This paper reports the long-term weight loss maintenance data following participation in the program.

**Methods:**

Of the 172 participants enrolled at the beginning of the study, data from 110 participants were available and analyzed at 24 months, i.e., 12 months after the end of the 12-month intervention.

**Results:**

No between-group difference in weight loss maintenance was observed. Pooled results showed a significant weight regain from 12 to 24 months, i.e., an average of 7.85 lbs. of the 20.36 lbs. lost. However, participants from both groups were still 12.51lbs or 6.13% lighter at 24 months than at baseline.

**Conclusion:**

If-then plans did not result in a higher percentage of weight loss at 24-month follow-up compared to the standard GLB. However, at 24 months, both groups did show a maintenance of a significant portion of the weight lost at the end of intervention.

**Trial registration:**

ClinicalTrials.gov Identifier: NCT02008435, registered 6 December 2013.

## Background

Behavior modification programs aimed at changing diet and physical activity have been shown to be an effective weight loss approach [1]. The most effective among these has been the one-on-one Diabetes Prevention Program (DDP) [2] for which clinically significant weight losses of 5–7% and a 58% lower incidence of diabetes compared to placebo have been found at an average 3 years post-intervention [3]. Participants in this program displayed a modest weight regain (e.g., 2.2 lbs.); however, the incidence of diabetes remained lower at 10-year follow-up [4].

Due to the high cost of the DPP, a group-based shortened version, called the Group Lifestyle Balance (GLB) program, was developed [5]. While less costly to implement and thus more accessible, its effectiveness is lower than that of the DPP. A recent meta-analysis showed that weight loss with the GLB was 3.99% at post-intervention compared to 7% with the DPP [6]. One of the few GLB intervention studies that provide longer term weight loss maintenance data found that the probability of achieving a 5% weight loss at 3 months was 45.7% (why the results were reported as probabilities and not actual percentages of weight loss is unclear), but only 17.3% of participants maintained this weight loss 1 year post-intervention [7]. Also, of those who lost at least 5% of their body weight post-intervention, 52.6% maintained it at 24 months, weighing approximately 20 lbs. less than at baseline [7].

To increase the weight maintenance effectiveness of the GLB, our study integrated habit formation techniques, specifically if-then plans, to reinforce habit change [8, 9]. We called this program the enriched GLB or the McGill Comprehensive Health Improvement Program (CHIP) Healthy Weight Program as it was conducted with the McGill CHIP, which is a multidisciplinary disease management and prevention program that is the primary focus of academic research and teaching activities on exercise and health for the McGill medical community. If-then plans (also called implementation intentions) are concrete contingency plans that specify when, where, and how to act in a specific situation [8, 9]. If-then plans have demonstrated medium-to-large effects in inducing habit change in a number of behaviors [10]. However, not many long-term studies of implementation intentions and complex behaviors yet exist.

The GLB standard and enriched program versions were delivered over 1 year (12 weekly core sessions, four transitional sessions over 3 months, and 6 monthly support sessions). The 3- (end of core sessions) and 12- (end of intervention) month results of the if-then enriched randomized controlled trial (RCT) have been published [11]. No between-group differences were found; that is, no apparent added benefit augmenting the GLB (control group) with the if-then plans (enriched GLB group) was observed. However, both groups displayed large weight losses of 9.98% over 12 months. One of the reasons hypothesized for the lack of significant group differences was that participants in both conditions were trained to become more aware of their internal and external cues and to create responses to these cues. As a consequence, the creation of if-then plans might have also occurred in the standard GLB ^group^ participants, just more implicitly. Also, the coaches in both groups were highly trained in cognitive behavioral therapy (CBT) [11] and spent two entire sessions teaching participants how to identify problematic cues and add positive food, physical activity, and social cues to their environment. Possibly, this behavioral training, in addition to the therapeutic contact with the coaches that all participants received, increased the overall effectiveness of the program but did not allow for much difference between the standard and enriched GLB groups.

This paper reports the findings of our RCT after a 1-year no-contact follow-up period, 24-months after baseline. We hypothesized that the if-then plans would serve as a protective barrier to weight regain. Specifically, we hypothesized that from the end of intervention (12 months) to follow-up (24 months), the if-then plan group would show greater weight loss maintenance than the group that was not explicitly instructed to create if-then plans.

## Methods

**Fig. 1:**
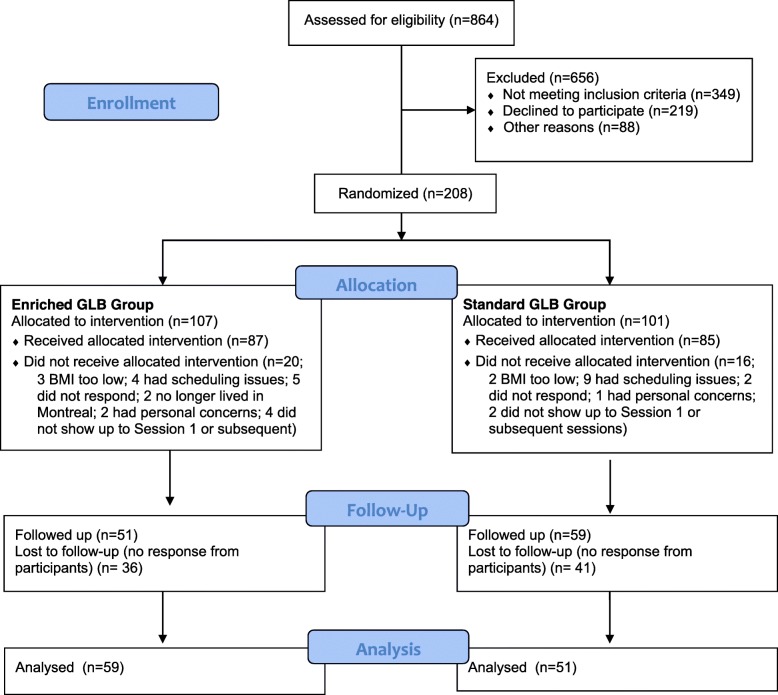
CONSORT flow diagram of the screening, group randomization, 24-month follow-up data, and analysis

The prospective, two-arm RCT was conducted between 2014 and 2017 with approval by the Research Ethics Board at McGill University (Montreal, Canada). A detailed description of the intervention, methods, procedures, and measures are published in the study protocol [12] and outlined in Fig. 1. Informed consent was obtained from all participants before any study procedures were conducted.

### Study procedures

The GLB manual was adhered to in both groups [5], and if-then plans were integrated into sessions of the enriched groups. Of the 172 participants who were enrolled at the beginning of the study, we were able to collect data from 110 participants at 24-month follow-up (64% retention; 51 in the enriched GLB group and 59 in the standard GLB group).

### Measures

The primary outcome, body weight, was assessed using a digital scale. Details of the measures of the secondary outcomes of goal achievement, diabetes risk factors, physical activity, self-monitoring, and habit strength are available in the study protocol [12]. Self-monitoring of food and exercise was completed through an online tracker. Habit strength was assessed using the Self-Report Index of Habit Strength [13].

### Statistical analyses

Study analyses were conducted using Mplus version 8.0 [14]. Multigroup analysis was used to examine change (i.e., mean difference) from the 12- to 24-month follow-up. As with the previous analysis [11], missing data for *N* = 62 participants were handled with the estimation procedure “use full information maximum likelihood” with robust standard errors. This allows all data to be included in the estimation [15, 16]. As such, the missing data were imputed internally in the same model examining change in weight over time. Missing weight measurements were then predicted from other weight measurements assessed at other time points. As Little’s missing completely at random test was not significant (*p* = 0.608), the missingness was assumed to follow a missing completely at random (MCAR) pattern. The full information maximum likelihood method performs equally well as listwise (or pairwise) deletion under MCAR [16]. The group difference was assessed using the rescaled −2 log likelihood difference test, which is distributed as chi-squared with degrees of freedom equal to the rescaled difference in the number of parameters between models. Specifically, the group difference was examined by comparing the fit of a model in which the change from 12 to 24 months was permitted to differ between groups with the fit of a model in which the change was restricted to be equal in both groups. A nonsignificant chi-square test value at *α* = .05 indicated no group difference in the estimate examined. Due to the nonsignificant model difference, average pooled change scores were computed across groups.

## Results

Demographic information of the participants at baseline and 24-month follow-up is presented in Table 1. Information about completed measures is reported in the protocol paper [12]. Mean changes in all study outcomes from 12 to 24 months are reported in Table 2. Chi-square values indicate that mean changes for all variables did not differ between groups.

**Table 1:** Demographic information of participants at baseline and 24-month follow-up

	Initially enrolled (*n* = 172)	Completed 24-month follow-up (*n* = 110)
Demographics		
Age, mean (*SD*) (years)	50.22 (11.97)	52.18 (11.65)
Gender, *n* (%) female	138 (80.23)	87 (79.09)
Caucasian, *n* (%)	135 (78.49)	90 (81.82)
Married, *n* (%)	99 (57.56)	65 (59.09)
Education, *n* (%) bachelor’s degree	124 (72.09)	79 (71.82)
Employed, *n* (%)	132 (76.74)	84 (76.36)
Household income, > $40,001, *n* (%)	117 (68.02)	75 (68.18)
Smoker, *n* (%)	9 (5.23)	3 (2.73)
Primary outcome		
Baseline weight, mean (*SD*) (lbs.)	204.03 (31.79)	202.16 (29.96)

**Table 2:** Mean changes in weight and secondary outcomes from 12 (post-intervention) to 24 months by group

	Standard GLB (*n* = 59)					Enriched GLB (*n* = 51)					*χ*^2^	*p*
	Mean (SE)	*Z*	*p*	*R*^2^	95% CI	Mean (SE)	*z*	*p*	*R*^2^	95% CI		
Primary outcome												
Weight (lbs.)	5.58 (5.26)	1.06	.289	0.01	−4.74, 15.89	10.78 (5.94)	1.81	.070	0.03	−0.87, 22.43	0.43	.514
Diabetes risk factors												
Waist circumference (cm)	−0.24 (2.46)	− 0.10	.923	0.00	−5.06, 4.59	1.00 (2.84)	0.35	.724	0.00	−4.57, 6.57	0.11	.742
Physical activity												
Physical activity total duration (min/week)	42.62 (49.17)	0.87	.386	0.01	− 53.75, 138.99	12.08 (97.05)	0.12	.901	0.00	− 178.15, 202.31	0.07	.788
Physical activity pedometer steps (per day)	993.27 (93.00)	1.07	.286	0.02	− 829.54, 2816.08	− 1131.73 (59.76)	−1.89	.058	0.06	− 2302.93, 394.70	3.21	.073
Physical activity step equivalents (per day)	1413.76 (158.14)	0.89	.371	0.01	− 1685.78, 4513.30	− 2210.32 (203.84)	−1.08	.278	0.02	− 6205.51, 1784.87	1.16	.282
Self-Monitoring index												
Food tracking frequency (days/week)	−0.61 (0.30)	−2.02	.044	0.02	−1.21, − 0.02	− 0.43 (0.22)	−1.90	.058	0.02	− 0.86, 0.01	0.25	.620
Activity tracking frequency (days/week)	−1.26 (0.35)	−3.59	<.001	0.07	−1.95, −0.57	−0.95 (0.34)	−2.85	.004	0.05	−1.61, −0.30	0.40	.528
Behavior change index												
Average fat intake (grams/day)	3.54 (3.13)	1.13	.258	0.01	−2.59, 9.67	2.34 (3.58)	0.65	.513	0.01	−4.67, 9.35	0.06	.801
Average caloric intake (per day)	18.85 (72.90)	0.26	.796	0.00	− 124.03, 161.73	−39.18 (80.68)	−0.49	.627	0.00	− 197.43, 118.95	0.29	.593
Habit strength index												
Total score	−0.46 (0.25)	−1.86	.063	0.03	−0.94, 0.03	−0.42 (0.23)	−1.81	.070	0.03	−0.88, 0.03	0.01	.910

### Primary outcome

Table 3 contains the means at baseline and 12 months and mean changes pooled across the two groups. Pooled results show a significant weight regain from 12 to 24 months (*p* = .047), with participants regaining on average 7.85 lbs. of the 20.36 lbs. that they had lost. However, participants were still 12.51 lbs. (or 6.13%) lighter at 24 months than at baseline; they had lost 9.98% of their initial body weight at the 12-month follow-up. More than half of all participants who achieved a clinically significant weight loss of 5% at post-intervention (62.07%) maintained this weight loss at 24 months.

**Table 3:** Mean changes in weight and secondary outcomes from 12 (post-intervention) to 24 months pooled across groups

	Mean at baseline	Mean at post-intervention (12 months)	Mean at follow-up (24 months)	Pooled Estimates (Change from 12 months to 24 months)					
				Mean change (SE)	*z*	*p*	95% CI	*R*^2^ *–* Standard GLB	*R*^2^ *–* Enriched GLB
Primary outcome									
Weight (lbs.)	204.03	183.67	191.52	7.85 (3.96)	1.98	.047*	0.09, 15.61	0.02	0.02
Diabetes risk factors									
Waist circumference (cm)	108.79	101.25	101.57	0.32 (1.87)	0.17	.863	−3.34, 3.98	0.00	0.00
Physical activity									
Physical activity total duration (min/week)	98.79	220.89	256.37	35.48 (45.37)	0.78	.434	−53.44, 124.40	0.01	0.00
Physical activity pedometer steps (per day)	7403.26	8740.25	8190.29	−549.96 (55.22)	−0.10	.319	− 1632.19, 532.26	0.01	0.02
Physical activity step equivalents (per day)	9058.36	13,369.17	11,724.57	− 1644.60 (165.78)	0.10	.921	− 3413.70, 3084.77	0.00	0.00
Self-Monitoring Index									
Food tracking frequency (days/week)	4.43	0.84	0.35	−0.49 (0.18)	−2.68	.007*	−0.85, − 0.13	0.02	0.03
Activity tracking frequency (days/week)	6.30	1.48	0.38	−1.10 (0.24)	−4.53	<.001*	−1.58, −0.62	0.06	0.06
Behavior Change Index									
Average fat intake (grams/day)	51.94	49.34	52.36	3.02 (2.36)	1.28	.201	−1.61, 7.64	0.01	0.01
Average caloric intake (per day)	1465.31	1434.85	1427.39	−7.46 (54.31)	−0.14	.891	−113.90, 98.97	0.00	0.00
Habit Strength Index									
Total score	2.85	4.41	3.97	−0.44 (0.17)	−2.60	.009*	−0.77, − 0.11	0.03	0.03

*Significant at *p* < 0.05

### Secondary outcomes

Pooled results showed significant changes at 24-month follow-up only for self-monitoring and habit strength. All other variables showed no significant change from 12 to 24 months; that is, all changes achieved at 12 months were maintained.

#### Self-Monitoring

Food tracking frequency significantly decreased for both groups from 12 months to 24 months by 0.49 days/week. Activity tracking frequency also decreased for both groups by 1.10 days/week. In comparison to their baseline values of 5 days/week for food tracking and 6 days/week for activity tracking frequency, both values were significantly lower at 24 months.

#### Habit strength

Habit strength showed a statistically significant decrease from 12 to 24 months by a mean score of 0.44. However, it remained significantly higher than the baseline total score of 2.

## Discussion

This paper reports the 24-month follow-up results of an intervention developed to increase and maintain weight loss of the GLB. Both groups showed long-term weight loss of about 6.13% compared to baseline, even though some weight regain occurred post-intervention. A weight loss of above 5% is considered to be clinically significant indicating an effective behavioral weight loss program [3]. Specifically, a 5–7% decrease in body weight has been shown to be linked to a 58% lower (compared to placebo) incidence of diabetes following participation in the DPP program [3]. However, in this study, no between-group differences were found. Furthermore, both groups showed significant decreases in self-monitoring and habit strength from 12 to 24 months, but habit strength remained higher than at baseline. No significant changes were seen after the end of the intervention for diabetes risk factor variables of weight circumference and physical activity duration, steps, and equivalents; that is, the positive changes that had accumulated in these variables at the end-of-intervention at 12 months remained at 24 months.

Pooled results over both conditions show that the long-term maintenance for our program was slightly better than the long-term maintenance in Piatt et al.’s study [7], which is the only study that provided long-term 24 month results for the standard GLB. That is, 62.07% of participants in our program versus 52.6% in Piatt et al.’s study maintained 5% weight loss at 24-month follow-up. Although both interventions were delivered in the community, ours was delivered by highly trained clinical psychology PhD students who were well versed in CBT and other behavior intervention strategies compared to delivery by lay coaches in the GLB study. In our study, possibly, the knowledge and experience of the coaches in facilitating behavior change improved its effectiveness, as explained above, and as such, the addition of explicit if-then planning was not a strong enough intervention to render stronger effects in the enriched GLB group compared with the standard GLB group. Further research assessing the effects of delivery method and coaches on GLB efficacy is needed.

A few limitations of the current study should be mentioned. Our 24-month results are compared only with one other study, namely that of Piatt et al. [7]. It is the only other currently available study that provides 24 months results for the GLB. Although a direct comparison must be made with caution, other behavioral weight loss program studies found that most people regain all of their weight by 6 to 12 months after completion of the lifestyle intervention and oftentimes even surpass their baseline weight [17]. Thus, the results of both our study and that of Piatt et al. shine an optimistic light at the ability of the GLB to effect clinically significant long-term maintenance.

The attrition rate at follow-up, which was 46% in this study (107 to 51 participants in the enriched GLB group and 101 to 59 in the standard GLB group), was another limitation of this study. However, the degree of attrition is typical for long-term follow-ups [18], and our statistical methods used to impute the missing data compensate for possible systematic attrition [15, 16].

The current study has implications for ways to achieve better long-term weight loss maintenance in group-based behavioral weight loss programs. Specifically, our study draws attention to the importance of training coaches to effectively teach behavior change techniques to participants. These techniques include if-then contingencies, which are an integral part of behavior planning and habit formation.

## Conclusion

We found large reductions in weight from baseline to 3 and 12 months for both the standard and the enriched GLB, with a significant portion maintained at 24 months. Participants lost 9.98% of their initial body weight at 12-month follow-up and retained this weight loss at 6.13% of their initial baseline weight at the 24-month follow-up. Furthermore, a greater percentage of those who lost 5% of their initial weight at the end of intervention maintained this weight loss at 24-month follow-up than in the most comparable study assessing weight loss maintenance of the standard GLB.

## Data Availability

The study protocol, statistical analysis plan, analytic code, and de-identified data that underlie the results from this study will be available at 3 months after article publication to researchers who provide a methodologically sound proposal. Proposals should be directed to the corresponding author. Data will be available following the signing of a data access agreement at the Open Science Framework (https://osf.io/).

## Abbreviations

CBT: Cognitive behavioral therapy
CHIP: Cardiovascular Health Improvement Program
DPP: Diabetes Prevention Program
MCAR: Missing completely at random
RCT: Randomized controlled trial
